# Tuberculosis control and economic recession: longitudinal study of data from 21 European countries, 1991–2012

**DOI:** 10.2471/BLT.14.142356

**Published:** 2015-04-15

**Authors:** Aaron Reeves, Sanjay Basu, Martin McKee, Andreas Sandgren, David Stuckler, Jan C Semenza

**Affiliations:** a Manor Road Building, Department of Sociology, University of Oxford, Oxford, OX1 3UQ, England.; b School of Medicine, Stanford University, Palo Alto, United States of America.; c Department of Public Health and Policy, London School of Hygiene & Tropical Medicine, London, England.; d European Centre for Disease Prevention and Control, Stockholm, Sweden.

## Abstract

**Objective:**

To investigate whether the economic recession affected the control of tuberculosis in the European Union.

**Methods:**

Multivariate regression models were used to quantify the association between gross domestic product, public health expenditure and tuberculosis case detection rates, using data from 21 European Union member states (1991–2012). The estimated changes in case detection attributable to the recession were combined with mathematical models of tuberculosis transmission, to project the potential influence of the recession on tuberculosis epidemiology until 2030.

**Findings:**

Between 1991 and 2007, detection rates for sputum-smear-positive tuberculosis in the European Union were stable at approximately 85%. During the economic recession (2008–2011) detection rates declined by a mean of 5.22% (95% confidence interval, CI: 2.54–7.90) but treatment success rates showed no significant change (*P* = 0.62). A fall in economic output of 100 United States dollars per capita was associated with a 0.22% (95% CI: 0.05–0.39) mean reduction in the tuberculosis case detection rate. An equivalent fall in spending on public health services was associated with a 2.74% (95% CI: 0.31–5.16) mean reduction in the detection rate. Mathematical models suggest that the recession and consequent austerity policies will lead to increases in tuberculosis prevalence and tuberculosis-attributable mortality that are projected to persist for over a decade.

**Conclusion:**

Across the European Union, reductions in spending on public health services appear to have reduced tuberculosis case detection and to have increased the long-term risk of a resurgence in the disease.

## Résumé

**Objectif:**

Enquêter pour déterminer si la récession économique a affecté la lutte antituberculeuse dans l'Union européenne.

**Méthodes:**

Des modèles de régression multivariée ont été utilisés pour quantifier l'association entre le produit intérieur brut, les dépenses de santé publique et les taux de détection des cas de tuberculose, à partir de données provenant de 21 États membres de l'Union européenne (période de 1991 à 2012). Les changements estimés dans la détection des cas de tuberculose imputables à la récession ont été combinés à des modèles mathématiques de transmission de la tuberculose afin de faire des projections concernant l'influence potentielle de la récession sur l'épidémiologie de la tuberculose jusqu'à 2030.

**Résultats:**

Entre 1991 et 2007, les taux de détection des cas de tuberculose à frottis d'expectoration positif dans l'Union européenne sont restés stables, à environ 85 %. Pendant la récession économique (de 2008 à 2011), les taux de détection ont baissé en moyenne de 5,22 % (intervalle de confiance de 95 %, IC : 2,54–7,90), mais les taux de réussite des traitements n'ont connu aucun changement significatif (P = 0,62). Une baisse de la production économique de 100 dollars US par habitant a été associée à une réduction moyenne du taux de détection des cas de tuberculose de 0,22 % (intervalle de confiance de 95 %, IC : 0,05–0,39 ). Une réduction équivalente dans les dépenses consacrées aux services de santé publique a été associée à une réduction moyenne du taux de détection de 2,74 % (intervalle de confiance de 95 %, IC : 0,31–5,16 ). Les modèles mathématiques suggèrent que la récession et les politiques d'austérité qui en ont résulté entraîneront des augmentations de la prévalence de la tuberculose et de la mortalité imputable à la tuberculose qui, d'après les projections réalisées, devraient durer pendant plus de dix ans.

**Conclusion:**

Dans l'Union européenne, les réductions des dépenses consacrées aux services de santé publique ont fait décliner la détection des cas de tuberculose et ont majoré le risque à long terme de résurgence de cette maladie.

## Resumen

**Objetivo:**

Investigar si la recesión económica ha afectado al control de la tuberculosis en la Unión Europea.

**Métodos:**

Se utilizaron modelos de regresión multivariados para cuantificar la asociación entre el producto interior bruto, el gasto en salud pública y las tasas de detección de casos de tuberculosis a partir de los datos de 21 estados miembro de la Unión Europea (1991 – 2012). Los cambios estimados en la detección de casos atribuibles a la recesión se combinaron con modelos matemáticos de transmisión de la tuberculosis para proyectar la influencia potencial de la recesión en la epidemiología de la tuberculosis hasta el 2030.

**Resultados:**

Entre 1991 y 2007, las tasas de detección de la tuberculosis con resultado positivo en el análisis de esputo en la Unión Europea se mantuvieron estables en torno al 85%. Durante la recesión económica (2008-2011), las tasas de detección se redujeron en un promedio del 5,22% (intervalo de confianza, IC, del 95%: 2,54-7,90), pero las tasas de éxito en el tratamiento no mostraron ningún cambio significativo (*P* = 0,62). Una caída en los resultados económicos de 100 dólares estadounidenses per cápita fue asociada a una reducción media del 0,22% (IC del 95%: 0,05-0,39) en la tasa de detección de casos de tuberculosis. Una caída equivalente en el gasto en los servicios de salud pública se asoció a una reducción media del 2,74% (IC del 95%: 0,31-5,16) en la tasa de detección. Los modelos matemáticos sugieren que la recesión y las políticas de austeridad consiguientes conducirán a un aumento de la prevalencia de la tuberculosis y de la mortalidad atribuible a esta enfermedad que se espera que persista durante más de una década.

**Conclusión:**

En la Unión Europea, los recortes en el gasto en servicios de salud pública parecen haber reducido la detección de casos de tuberculosis y haber incrementado el riesgo a largo plazo de un resurgimiento de la enfermedad.

## ملخص

الغرض استقصاء ما إذا كان للركود الاقتصادي تأثير على مكافحة السل في الاتحاد الأوروبي.

الطريقة استخدمت نماذج تحوّف متعددة المتغيرات لتحديد العلاقة الكمية ما بين الناتج المحلي الإجمالي، والإنفاق على الصحة العمومية، ومعدلات اكتشاف حالات السل، وذلك باستخدام بيانات مأخوذة من 21 من الدول الأعضاء في الاتحاد الأوروبي (في الفترة ما بين عام 1991 إلى عام 2012). تم دمج التغيرات المقدرة في اكتشاف الحالات التي تعزى إلى الركود مع النماذج الرياضية لسراية السل، لإبراز التأثير المحتمل للركود في مجال وبائيات السل حتى عام 2030.

النتائج في الفترة ما بين عامي 1991 و2007، كانت معدلات اكتشاف السل الإيجابي للطاخة - البلغم في الاتحاد الأوروبي مستقرة عند نسبة 85% تقريبًا. خلال الركود الاقتصادي (في الفترة ما بين عام 2008 إلى عام 2011)، انخفضت معدلات الاكتشاف بنسبة بلغت 5.22% في المتوسط (بنسبة أرجحية مقدارها 95%: 2.54–7.90) ولكن لم تظهر معدلات نجاح العلاج أي تغير يُعتد به إحصائيًا (الاحتمال = 0.62). وارتبط هبوط الحاصل الاقتصادي بمقدار 100 دولار أمريكي للفرد الواحد مع متوسط انخفاض يبلغ 0.22% (بنسبة أرجحية مقدارها 95%: 0.05–0.39) في معدلات اكتشاف حالات السل. ارتبط الهبوط المكافئ في الإنفاق على الخدمات الصحية العمومية بانخفاض بلغت نسبته في المتوسط 2.74% (بنسبة أرجحية مقدارها 95%: 0.31–5.16) في معدلات الاكتشاف. تشير النماذج الرياضية إلى أن الركود وما ينشأ عنه من سياسات تقشف الناشئة ستؤدي إلى زيادات في معدلات انتشار السل والوفيات التي تعزى إلى السل والتي من المتوقع أن تستمر لأكثر من عقد.

الاستنتاج في جميع أنحاء الاتحاد الأوروبي، يبدو أن انخفاض الإنفاق على الخدمات الصحية العمومية له تأثير في انخفاض اكتشاف حالات السل وزيادة الخطر من عودة ظهور المرض على المدى الطويل.

## 摘要

**目的:**

旨在调查欧盟国家的经济衰退是否会影响对结核病的控制。

**方法:**

基于从 21 个欧盟成员国采集的数据(1991 – 2012 年),多元回归模型用以量化国内生产总值、公共卫生支出和结核病例检测率之间的联系。因经济衰退引起的发病病例中的预估变化与结核病传染的数学模型结合起来,可预测出经济衰退在 2030 年前对结核病流行病学的潜在影响。

**结果:**

在 1991 年至 2007 年期间,欧盟国家的痰涂片阳性结核病检测率大概稳定在 85%。在经济衰退(2008 – 2011 年)期间,检测率平均下降了 5.22%(95% 置信区间,CI:2.54 - 7.90),但是治疗成功率并无显著变化 (*P* = 0.62)。人均经济产量降低 100 美元与结核病例检测率平均下降 0.22% (95% CI:0.05 - 0.39) 相关。公共卫生服务方面的支出降低同等金额与检测率平均下降 2.74% (95% CI:0.31 – 5.16) 相关。数学模型表明,经济衰退和随之而来的紧缩政策将导致结核病发病率增加,而由结核病引起的死亡率预计会持续十多年的时间。

**结论:**

在整个欧盟中,公共卫生服务的支出减少导致结核病例的检测率降低,从而促使疾病复发的长期风险提高。

## Резюме

**Цель:**

Определить, повлиял ли экономический спад на борьбу с туберкулезом в Европейском союзе.

**Методы:**

Использовались многомерные регрессионные модели для количественной оценки связи между объемом валового внутреннего продукта, расходами на здравоохранение и показателями выявления туберкулеза на основе данных из 21 страны-члена Европейского союза (1991–2012 гг.). Оцененные изменения в количестве выявляемых случаев заболевания, связанные с экономическим спадом, были скомбинированы с математическими моделями распространения туберкулеза с целью определения потенциального влияния экономического кризиса на эпидемиологию туберкулеза до 2030 года.

**Результаты:**

В 1991–2007 гг. показатель обнаружения туберкулеза легких у мокротопозитивных больных в Европейском союзе был стабильным и составлял примерно 85%. Во время экономического спада (2008–2011 гг.) уровень обнаружения снизился в среднем на 5,22% (95% доверительный интервал, ДИ: 2,54–7,90), но при этом процент успеха в лечении существенно не изменился (*P* = 0,62). Падение объемов производства в размере 100 долларов США на душу населения было связано со средним снижением показателя частоты выявления случаев туберкулеза, составившим 0,22% (95% ДИ: 0,05–0,39). Аналогичное падение расходов на здравоохранение было связано со средним снижением показателя выявления, составившим 2,74% (95% ДИ: 0,31–5,16). Математические модели показывают, что экономический спад и последующая политика жесткой экономии приведут к увеличению распространенности туберкулеза и смертности от этого заболевания. Согласно прогнозам, это увеличение продлится в течение более десяти лет.

**Вывод:**

Сокращение расходов на здравоохранение в Европейском союзе, по-видимому, повлияло на уменьшение показателя выявления случаев туберкулеза и увеличение долгосрочного риска, связанного с возобновлением высокого уровня заболеваемости.

## Introduction

Tuberculosis control requires a strong public health infrastructure to detect and treat infected people.^1^^–^^8^ The World Health Organization (WHO) has identified improved case detection and successful treatment as priority actions required to meet target levels of tuberculosis prevention and control by 2015.^9^ Such improvements will require expanding surveillance and diagnosis services – especially among more vulnerable groups.^10^^–^^13^ Over the past two decades, global rates of case detection and treatment success for tuberculosis have risen steadily.^14^ However, detection and treatment remain poor in several countries, mostly in eastern Europe and sub-Saharan Africa.^15^

Western Europe has high rates of active case detection and treatment success.^9^ However, the 2008–2011 economic recession and resulting cuts in health budgets may have weakened tuberculosis control and prevention programmes.^16^ Economic recessions are often accompanied by increases in drug use, homelessness, migration of vulnerable groups and other factors affecting the transmission of tuberculosis.^17^ In a scoping study, 27 infectious disease experts predicted that understaffing, recruitment freezes and reductions in the workforce during the recession in Europe that began in 2008 would have a negative regional impact on the control and treatment of various communicable diseases.^18^ Tuberculosis was the disease most commonly cited as a cause for concern. 

Despite these concerns, several member states of the European Union have introduced user fees or budget cuts to infectious disease programmes since the onset of the recession. Between 2008 and 2010, for example, Latvia shifted approximately 50% of the costs of diagnostic testing to patients and reduced spending on disease control and surveillance by 87%.^19^ Charges for prescription drugs were also increased in Ireland in 2009–2010.^19^ A recession may worsen the negative effect of payments for diagnostic tests or treatment.^20^^,^^21^ However, not all European countries reduced funding for communicable disease programmes. Estonia reduced health-care spending after the recession began but protected spending on the detection of communicable diseases. Croatia reduced user charges for prescription medication by 33% and both Austria and Germany increased their budgets for infectious disease prevention and control.^16^

Here, we test the hypothesis that the recent economic recession and associated reductions in public health spending resulted in declining rates of case detection and treatment success for tuberculosis in the European Union. We then use mathematical models that account for the nonlinear dynamics of tuberculosis, to simulate the consequences of economic changes on the future trends in tuberculosis incidence, prevalence and mortality.

## Methods

### Data sources

Data on tuberculosis case detection and treatment success rates were taken from the 2014 edition of the WHO’s tuberculosis database.^22^ Data on total health spending, expenditure on public health services and gross domestic product (GDP) were taken from the EuroStat database.^23^ All macroeconomic data were analysed as per capita values and adjusted for inflation and purchasing power to facilitate comparisons across member states of the European Union. At the time of our analysis, data on public health spending were available for 24 of the 28 member states. Data were not available for Belgium, Greece, Romania and Slovakia because these member states either lack a specific budget line or do not report disaggregated expenditure data to EuroStat. As we excluded Cyprus, Luxembourg and Malta because of their small population sizes, most of our final analyses were based on the data from 21 member states (available from corresponding author).

We estimated case detection rates as the proportion of annual tuberculosis incidence that was reported in case notification data.^22^ The gap between those cases that are notified and those that are not – because they are not diagnosed or are diagnosed but not reported – represents the underreporting of incidence. The estimated rate of case detection may exceed 100% if the true incidence is underestimated and/or if cases are double-counted.^24^ Despite such issues, the case detection rate remains one of the most widely used indicators of progress in establishing effective tuberculosis control.^1^^,^^9^

For our analysis, a person with tuberculosis who completed a full course of treatment was considered to be a treatment success – whether there was evidence of a cure or not. We measured rates of treatment success as percentages of (i) new sputum-smear-positive cases, (ii) other new cases of tuberculosis – i.e. new extrapulmonary cases and new pulmonary cases who had been found sputum-smear-negative or did not have a sputum-smear result, and (iii) re-treatment cases.

### Statistical models

In the first step of our analysis, we quantified the extent to which rates of tuberculosis case detection and treatment success changed across the European Union, using equation (1):
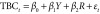
(1)where TBC represents either the case detection rate or treatment success rate, which were estimated as separate models, *β* is a regression coefficient, *t* is the year, *Y* is the linear time trend in the case detection rate across the European Union, *R* is a binary indicator marking the European Union’s recession (2008–2011) and *ε* is an error term. Case detection rates for the previous 12 months were based on the estimated proportion of new smear-positive cases that had been detected across all 21 of our study countries. Treatment success rates were estimated separately for smear-negative, smear-positive and re-treatment cases of tuberculosis.

In a subsequent step, we tested whether the economic downturn and/or reductions in public health expenditure could account for the observed changes in rates of tuberculosis case detection over time, using equation (2):
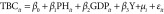
(2)where *i* is a country, PH is government expenditure on public health services per capita, GDP is a measure of the GDP per capita and *µ* is the country fixed effect. In a third step, the severity of recession was based on the cumulative decline in GDP for each country during the recession. For all of our econometric models, we adjusted for time trends and country-specific fixed effects. Models were investigated using Stata version 13 (StataCorp. LP, College Station, United States of America).

### Mathematical models and simulations

To forecast tuberculosis incidence, prevalence and mortality in each of our study countries, we applied the findings from the preceding econometric models to dynamic mathematical models of tuberculosis transmission and mortality. The mathematical models simulated longitudinal tuberculosis rates in each country – given the data on case detection observed before, during and after the financial crisis – as well as a counterfactual scenario in which case detection was unaffected by either the recession or the related austerity.

The modelling approach we followed was derived from standardized models that are commonly used in tuberculosis modelling and have been described elsewhere.^25^^–^^30^ Briefly, we included conditions of susceptibility to tuberculosis, recent latent infection, remote latent infection, active smear-positive tuberculosis, active smear-negative or extrapulmonary tuberculosis and recent recovery from tuberculosis. We used a Markov chain, Monte Carlo algorithm to simulate transmission of tuberculosis within each of our study countries. For calibration, we used the corresponding longitudinal trajectory seen in tuberculosis incidence, prevalence and mortality between 1990 and 2012 (available from corresponding author). While inputting the case detection and treatment success rates observed for each country, we fitted the corresponding transmission rate and time between symptom onset and case detection to the longitudinal trajectory of tuberculosis incidence, prevalence and mortality from 1990 to 2012 – ensuring an error of less than 5% between the model and observed data. We then compared the observed scenario in which case detection rates dropped during austerity – including the modelled tuberculosis outcomes for 2013–2030 – with the counterfactual scenario in which case detection rates followed the same linear trends as those that occurred before the onset of the recession.

We used sensitivity and uncertainty analyses to examine the robustness of the modelled results.

## Results

### Cross-national trends

**Fig. 1:**
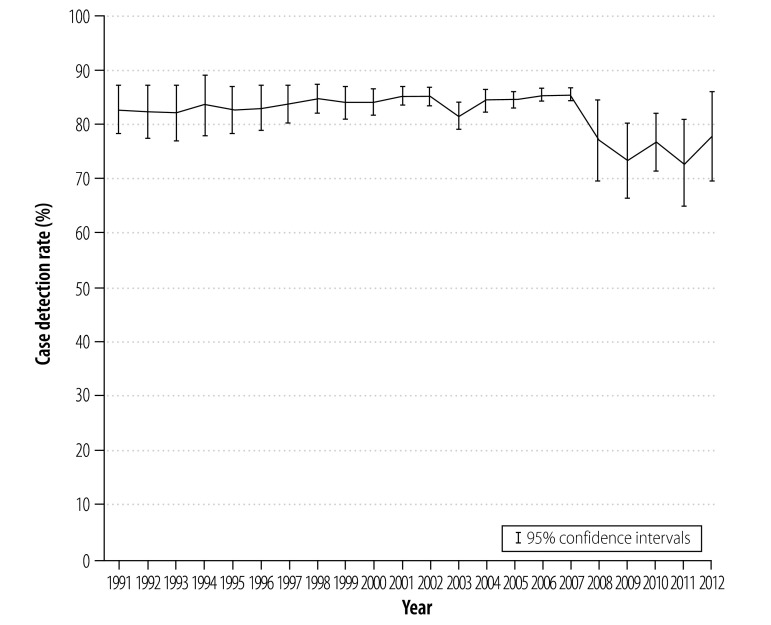
Trends in rates of tuberculosis case detection, European Union, 1991–2012

Note: The relevant data for Luxembourg were not available. Data source: World Health Organization.^22^

**Fig. 2:**
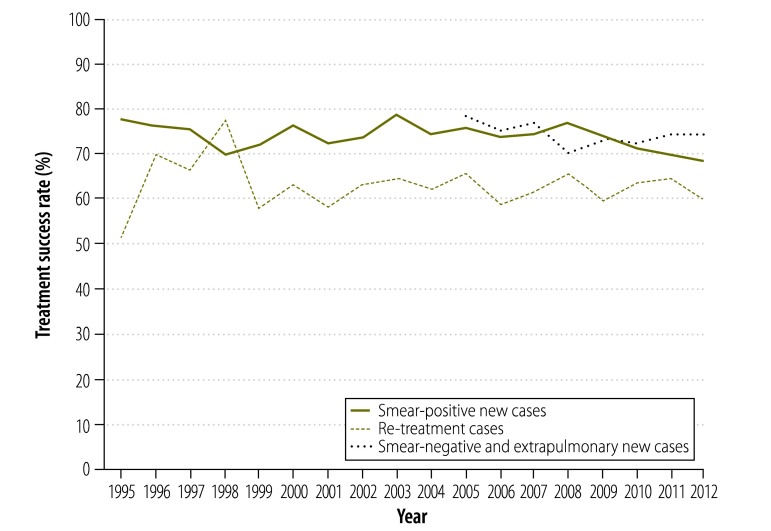
Trends in rates of tuberculosis treatment success, European Union, 1995–2012

Before the recession – between 1991 and 2007 – case detection rates were approaching 85% across the European Union. Subsequently the rate of case detection fell (Fig. 1). We estimated that case detection rates declined by a mean of 5.22% (95% confidence interval, CI: 2.54–7.90) during the recession (Table 1). In contrast, treatment success rates appear to have remained stable in the European Union (Fig. 2). As the recession had no observable effect on treatment success rates among smear-negative, smear-positive or re-treatment cases, the remainder of this paper focuses on variations in case detection rates.

**Table 1:** Trends in tuberculosis case detection and treatment success rates, European Union, 1991–2012

Time period	Change in case detection rate, % (SE)	Change in treatment success rate		
		Smear-negative and extrapulmonary new cases, % (SE)	Smear-positive new cases, % (SE)	Re-treatment cases, % (SE)
Annual trend	−0.22 (0.12)	−0.56 (0.58)	−0.21 (0.40)	−0.10 (0.50)
2008–2011	−5.22 (1.24)**	0.94 (2.66)	2.15 (2.96)	1.57 (3.68)

SE: standard error. ** *P* < 0.01.

Notes: Data represent the combined values for 21 countries – i.e. all of the member states of the European Union in 2014 excluding Belgium, Cyprus, Greece, Luxembourg, Malta, Romania and Slovakia. Data on case detection rates and treatment success rates were available among smear-negative patients for the years 1995–2012, extrapulmonary new cases for the years 2004–2012, smear-positive new cases for the years 2000–2012 and re-treatment cases for the years 2000–2012.

Data source: World Health Organization^22^and EuroStat.^23^

Data source: World Health Organization tuberculosis database.^22^

### Effects of recession

To assess the effect of the recession on case detection, we evaluated two standard indicators of an economic downturn: the annual changes in GDP per capita (model 1, Table 2), and a measure of the severity of the recession – which was based on the cumulative declines in GDP per capita for each country (model 2, Table 2).^16^^,^^31^ Annual economic growth appeared to have no effect on detection rates (*P* = 0.60), but cumulative declines in GDP during the recession were associated with falling case detection rates. A cumulative fall in GDP per capita of 100 United States dollars (US$) was associated with a reduction of 0.22% (95% CI: 0.05–0.39) in the detection rate (Table 2).

**Table 2:** GDP per capita and tuberculosis case detection rates, European Union, 1991–2012

Covariate	Change in case detection rate	
	Model 1, % (SE)	Model 2, % (SE)
Decline in annual GDP per capita of US$ 100	−0.79 (3.22)	NA
Cumulative decline in GDP per capita of US$ 100 (2008–2011)	NA	−0.22 (0.08)*

GDP: gross domestic product; NA: not included in model; SE: standard error; US$: United States dollars. * *P* < 0.05.

Notes*:* Data for 21 countries – i.e. all of the member states of the European Union in 2014 except Belgium, Cyprus, Greece, Luxembourg, Malta, Romania and Slovakia – and 316 country-years. Standard errors were adjusted for repeated observations within countries. Adjusted for country differences that were constant over time and for linear time trends.

Data source: World Health Organization^22^and EuroStat.^23^

**Fig. 3:**
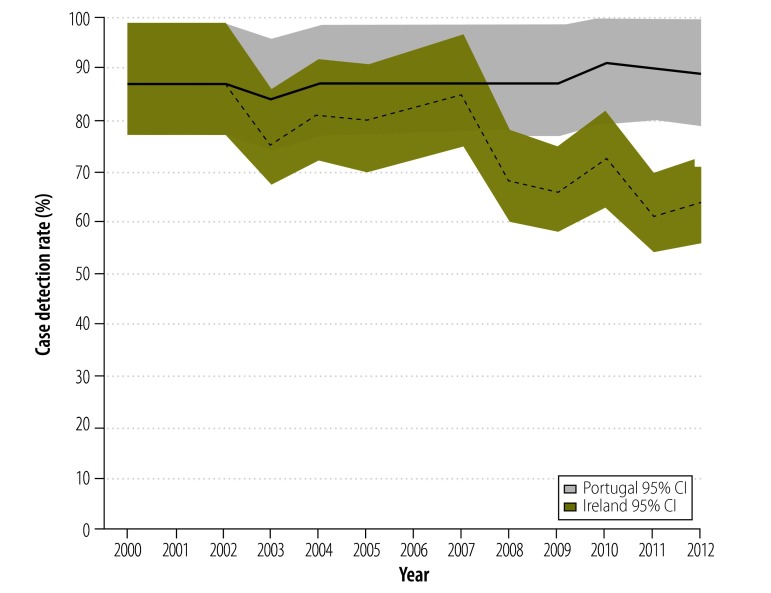
Rates of tuberculosis case detection, Ireland and Portugal, 2000–2012

**Fig. 4:**
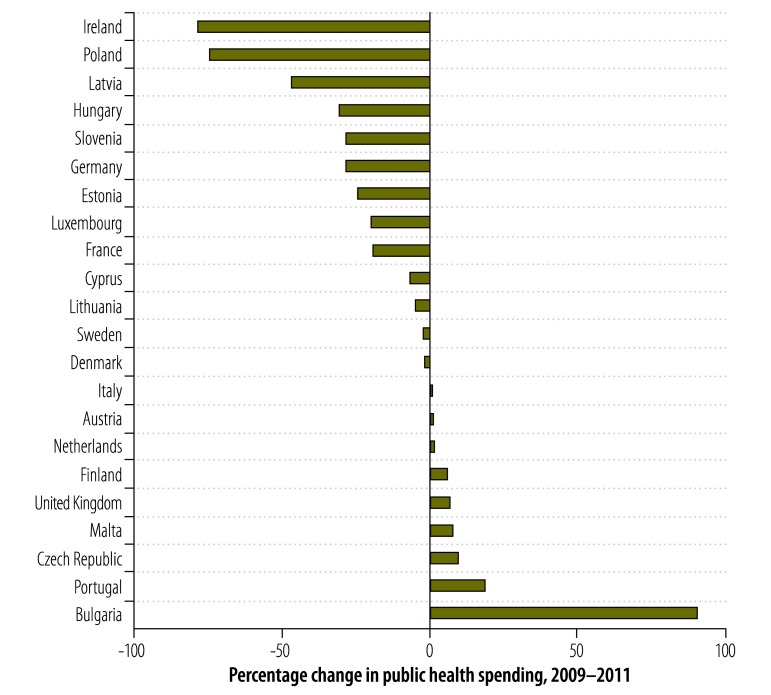
Expenditure on public health services, European Union, 2009–2011

Note: The relevant data for Belgium, Greece, Romania, Slovakia and Spain were not available.

Case detection rates did not decline in every country that experienced a recession. Although both Ireland and Portugal experienced deep recessions, the tuberculosis case detection rate fell in Ireland but not in Portugal (Fig. 3). Ireland implemented substantial reductions in public health spending whereas Portugal reduced its total government expenditure but initially protected its spending on public health services (Fig. 4).

CI: confidence interval.

Data source: World Health Organization.^22^

Data source: EuroStat.^23^

To test the role of budgetary decisions further, we included government expenditure on public health services in the statistical models (model 3, Table 3). Each US$ 100 decline in spending on public health services was associated with a decline of 3.11% (95% CI: 0.68–5.53) in the case detection rate. The magnitude of this effect was not significantly changed after adjusting for fluctuations in GDP per capita (*β* = 3.18%; 95% CI: 0.88–5.48) or for cumulative declines in GDP per capita (*β* = 2.74%; 95% CI: 0.31–5.16).

**Table 3:** Expenditure on public health and tuberculosis case detection rates, European Union, 1991–2012

Covariate	Change in case detection rate		
	Model 1, % (SE)	Model 2, % (SE)	Model 3, % (SE)
Increase in public health spending of US$ 100 per capita	3.11 (1.16)*	3.18 (1.10)**	2.74 (1.16)*
Fall in annual GDP per capita of US$ 100	NA	−1.21 (3.06)	NA
Cumulative fall in GDP per capita of US$ 100 during recessionary years of 2008–2011	NA	NA	−0.21 (0.08)*

GDP: gross domestic product; NA: not included in model; SE: standard error; US$: United States dollars. * *P* < 0.05; ** *P* < 0.01.

Notes*:* Data for 21 countries – i.e. all of the member states of the European Union in 2014 except Belgium, Cyprus, Greece, Luxembourg, Malta, Romania and Slovakia – and 316 country-years. Standard errors were adjusted for repeated observations within countries. Adjusted for country differences that were constant over time and for linear time trends.

Data source: World Health Organization^22^and EuroStat.^23^

### Role of underreporting

**Fig. 5:**
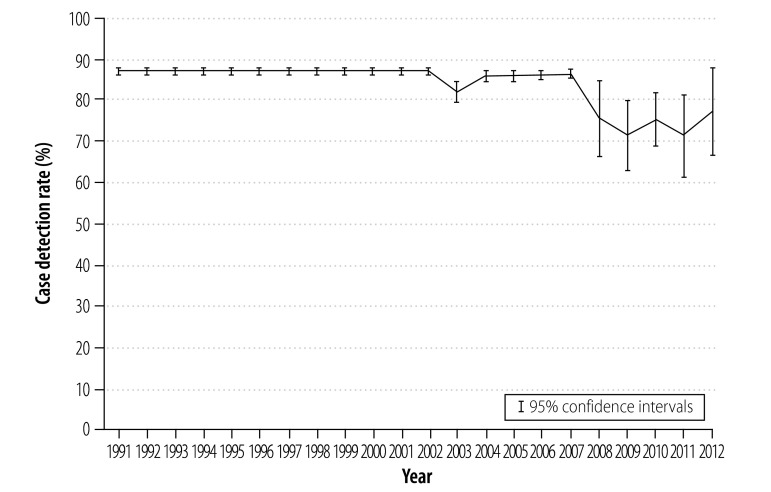
Rates of tuberculosis case detection in countries with apparently stable levels of underreporting, European Union, 1991–2012

Note: Bulgaria, Hungary, Latvia, Lithuania, Luxembourg and Romania were excluded because they reported varying levels of underreporting over the study period.

Since case detection reflects the gap between case notifications and estimated incidence, a rise in underreporting – which may have resulted from cuts in surveillance systems – may have reduced estimates of case detection rates. To test this possibility, we excluded Bulgaria, Hungary, Latvia, Lithuania and Romania from the analysis because they reported changes in underreporting across the study period. Compared with the full analysis, this analysis indicated a stronger association between public health spending and the tuberculosis case detection rates (*β* = 3.53%; 95% CI: 1.12–5.94; Table 4 and Fig. 5).

**Table 4:** Expenditure on public health and tuberculosis case detection rates, European Union, 1991–2012, in countries where underreporting of cases appeared stable

Covariate	Change in case detection rate		
	Model 1, % (SE)	Model 2, % (SE)	Model 3, % (SE)
Increase in public health spending of US$ 100 per capita	3.53 (1.14)**	3.54 (1.12)**	3.05 (1.13)*
Decline in annual GDP per capita of US$ 100	NA	0.44 (3.60)	NA
Cumulative decline in GDP per capita of US$ 100 during recessionary years of 2008–2011	NA	NA	−0.26 (0.09)*

GDP: gross domestic product; NA: not included in model; SE: standard error; US$: United States dollars. * *P* < 0.05; ** *P* < 0.01.

Notes*:* Data for 17 countries: all of the member states of the European Union in 2014 except the countries with varying levels of underreporting – i.e. Bulgaria, Hungary, Latvia, Lithuania and Romania – and Belgium, Cyprus, Greece, Luxembourg, Malta and Slovakia. They also represent 267 country-years. Standard errors were adjusted for repeated observations within countries. Adjusted for country differences that were constant over time and for linear time trends.

Data source: World Health Organization tuberculosis database^22^and EuroStat.^23^

Data source: World Health Organization.^22^

### Tuberculosis forecasting

**Fig. 6:**
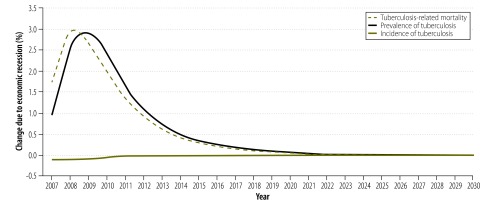
Simulating tuberculosis transmission and mortality, European Union, 2007–2030

Notes: One model was used to simulate a crisis scenario in which, as generally observed, the budgetary austerity led to reduced rates of tuberculosis case detection. The other model simulated a non-crisis or counterfactual scenario in which case detection in each country was unaffected by the financial crisis and continued to follow the secular trend observed before the recession began. The plotted lines indicate the differences between the two models that can be attributed to budgetary austerity.

To understand the complex longer-term effects of the changes in case detection associated with the recession on overall tuberculosis trajectories, we used the results of our econometric analysis as inputs in a dynamic mathematical model of tuberculosis transmission and mortality. Fig. 6 shows the effect of recession and austerity compared with a counterfactual of continued economic growth. Further sensitivity analyses around the parameter values did not change these findings substantially.

### Robustness of models

Adjustment for nonlinear time trends using dummy variables for year attenuated the impact of GDP – but not that of public health expenditure – on case detection (Table 5). Using treatment success rates as the dependent variable, we repeated the statistical models. We found no significant association between treatment success and GDP or public health spending (Table 6). After adjusting for all other forms of health expenditure, we found that the effect of public health expenditure on the tuberculosis case detection rate was attenuated slightly but remained statistically significant (Table 7). As tuberculosis in Europe is partly fuelled by migration, changes in reported tuberculosis cases may be attributable to population movement rather than to changes in case detection. Unfortunately, the data available on tuberculosis trends in migrants to the European Union are problematic as they often lack accurate denominators for the migrants’ countries of origin.^32^ Adjustment of our main models for changes in overall levels of immigration during our study period did not affect our main findings (Table 8).

**Table 5:** Expenditure on public health and tuberculosis case detection rates, European Union, (1991–2012): effect of adding dummy variables for year

Covariate	Change in case detection rate		
	Model 1, % (SE)	Model 2, % (SE)	Model 3, % (SE)
Increase in public health spending of US$ 100 per capita	3.21 (1.12)**	3.47 (0.87)**	3.21 (1.17)*
Decline in annual GDP per capita of US$ 100	NA	4.64 (4.42)	NA
Cumulative decline in GDP per capita of US$ 100 during recessionary years of 2008–2011	NA	NA	0.01 (0.20)

GDP: gross domestic product; NA: not included in model; SE: standard error; US$: United States dollars. * *P* < 0.05; ** *P* < 0.01.

Notes*:* Data for 21 countries – i.e. all of the member states of the European Union in 2014 except Belgium, Cyprus, Greece, Luxembourg, Malta, Romania and Slovakia – and 316 country-years. Standard errors were adjusted for repeated observations within countries. Adjusted for country differences that were constant over time and for linear time trends.

Data source: World Health Organization^22^and EuroStat.^23^

**Table 6:** Public health services, GDP per capita and treatment success rates for tuberculosis, European Union, 1991–2012

Covariate	Change in treatment success rate		
	Model 1, % (SE)	Model 2, % (SE)	Model 3, % (SE)
**Smear-negative and extrapulmonary new cases**^a^			
Increase in public health spending of US$ 100 per capita	4.24 (3.72)	4.34 (3.50)	4.34 (3.48)
Decline in annual GDP per capita of US$ 100	NA	0.02 (0.05)	NA
Cumulative decline in GDP per capita of US$ 100 during recessionary years of 2008–2011	NA	NA	0.08 (0.13)
**Smear-positive new cases**^b^			
Increase in public health spending of US$ 100 per capita	0.27 (0.57)	0.37 (0.59)	0.67 (0.75)
Decline in annual GDP per capita of US$ 100	NA	0.04 (0.05)	NA
Cumulative decline in GDP per capita of US$ 100 during recessionary years of 2008–2011	NA	NA	0.18 (0.13)
**Re-treatment cases**^c^			
Increase in public health spending of US$ 100 per capita	3.22 (3.53)	3.14 (3.50)	3.32 (3.61)
Decline in annual GDP per capita of US$ 100	NA	−0.02 (0.07)	NA
Cumulative decline in GDP per capita of US$ 100 during recessionary years of 2008–2011	NA	NA	0.04 (0.13)

GDP: gross domestic product; NA: not included in model; SE: standard error; US$: United States dollars.

^a^ Based on data from 18 countries and 126 country-years.

^b^ Based on data from 19 countries and 216 country-years.

^c^ Based on data from 19 countries and 201 country-years.

Notes*:* Standard errors were adjusted for repeated observations within countries. Adjusted for country differences that were constant over time and for linear time trends.

Data source: World Health Organization tuberculosis database^22^and EuroStat.^23^

**Table 7:** Expenditure on public health, other government health spending, and tuberculosis detection rates, European Union, 1991–2012

Covariate	Change in case detection rate	
	Model 1, % (SE)	Model 2, % (SE)
Increase in public health spending of US$ 100 per capita	3.11 (1.16)*	2.62 (1.03)*
Increase in government health spending, excluding public health, of US$ 100 per capita	NA	−0.54 (0.42)

NA: not included in model; SE: standard error; US$: United States dollars. * *P* < 0.05.

Notes*:* Data for 21 countries – i.e. all of the member states of the European Union in 2014 except Belgium, Cyprus, Greece, Luxembourg, Malta, Romania and Slovakia – and 316 country-years. Standard errors were adjusted for repeated observations within countries. Adjusted for country differences that were constant over time and for linear time trends.

Data source: World Health Organization tuberculosis database^22^and EuroStat.^23^

**Table 8:** Effect of adjusting for immigration in estimating the effects of changes in public health spending on tuberculosis case detection rates, European Union, 1991–2012

Covariate	Change in case detection rate		
	Model 1, % (SE)	Model 2, % (SE)	Model 3, % (SE)
Increase in public health spending of US$ 100 per capita	3.11 (1.16)*	2.94 (1.10)*	2.73 (1.26)*
Decline in annual GDP per capita of US$ 100	NA	−0.04 (3.98)	NA
Cumulative decline in GDP per capita of US$ 100 during recessionary years of 2008–2011	NA	NA	−0.22 (0.08)*
1% increase in immigration	NA	1.58 (1.20)	1.21 (1.15)

GDP: gross domestic product; NA: not included in model; SE: standard error; US$: United States dollars. * *P* < 0.05.

Notes*:* Data for 21 countries – i.e. all of the member states of the European Union in 2014 except Belgium, Cyprus, Greece, Luxembourg, Malta, Romania and Slovakia – and either 316 country-years (model 1) or 245 country-years (models 2 and 3). Standard errors were adjusted for repeated observations within countries. Adjusted for country differences that were constant over time and for linear time trends.

Data source: World Health Organization tuberculosis database^22^and EuroStat.^23^

## Discussion

Rates of tuberculosis case detection fell by about 5% across the European Union during 2008–2011. This reduction was significantly linked to the economic recession and to reductions in public health spending. Using mathematical models, we estimated that the combined shocks of a recession and reductions in the budgets available for case detection would increase tuberculosis prevalence and tuberculosis-attributable mortality by as much as 3% for more than a decade after the recession.

Recession and economic austerity would be expected to lower case detection rates and therefore lead to an increase in tuberculosis-attributable mortality – since fewer tuberculosis patients would be detected and effectively treated. In consequence, fewer patients would live long enough to be able to relapse from recovery to active tuberculosis or to produce secondary incident cases and tuberculosis incidence would be relatively low. However, any short-term decline in incidence would soon be replaced by a rise in reported incidence as more – undetected – latent cases infect others. In the long term, the rise in prevalent cases would lead to higher incidence.

Our study has several important limitations. First, missing data meant that we had to exclude Greece – and some other European countries that reduced health-care spending during the recession – from our analysis.^33^ However, exclusion of these countries probably led to the associations we observed appearing weaker than they might otherwise have done. Second, as case detection rates are estimated as the ratio between case notifications and the estimated incidence, our main dependent variable may have been affected by measurement error. However, errors in the estimation of underreporting should not bias our attempts to observe potential associations between short-term economic changes and fluctuations in the rates of the detection and successful treatment of tuberculosis. When we excluded those countries where estimated levels of underreporting were known to have fluctuated over time, our main findings remained unaltered. Third, the indicator we used for public health expenditure was not restricted to tuberculosis-related expenditure but also included spending on the prevention of other diseases and the operation of other health services. Again, this limitation may have reduced the apparent strength of the observed associations. Fourth, the WHO’s estimates of tuberculosis prevalence are revised annually and may be subject to retrospective adjustment– but any changes are unlikely to alter the main implications of our findings. Fifth, while changes in diagnostic practices in some countries – e.g. moving towards methods of laboratory confirmation other than smear positivity – may influence tuberculosis case detection rates, such changes are generally slow and unlikely to create short-term fluctuations in case detection. Sixth, reductions in public health expenditure did not explain all of the variation in case detection rates. One other possible explanation is that case detection fell as a result of delays in seeking treatment – delays that were not captured in our statistical models.^24^ Future research is needed to understand the policy decisions behind the cuts we observed in public health expenditure and to link them to changes in case detection and other health outcomes.^18^^,^^19^ Reductions in public health expenditure have often been coupled with erosion of social safety nets.^34^^,^^35^ More work is needed to understand how these aspects of austerity may affect tuberculosis incidence and treatment success.^15^^,^^36^^–^^38^

Mathematical models revealed the potential short- and long-term effects of recessions and austerity on tuberculosis case detection. We found that inadequate detection and treatment can lead, in the short-term, to declines in the number of people alive to relapse or transmit the disease – artificially deflating the incidence. However, if low rates of case detection persist, new prevalent cases can become more abundant and increase tuberculosis incidence. High incidence can then lead to elevated case-loads and mortality levels that can persist for over a decade beyond the period of recession and austerity.

Our simulation models cannot capture all of the complex changes that occur during a recession. As crowding and other behaviours that increase contact rates may become more frequent during recessions, our model of transmission may underestimate the impact of recession and/or budgetary austerity on tuberculosis outcomes.

Tuberculosis control is sometimes seen as a soft target for spending cuts.^39^ Many of the infectious disease experts that we interviewed in a previous study were concerned that European governments would focus any recession-related reductions in health spending on tuberculosis control initiatives.^18^ Indeed, this concern has been borne out as some governments have substantially reduced their spending on public health services and communicable disease control in recent years.

Declines in the case detection rate cause delays in tuberculosis detection and treatment. The European Centre for Disease Prevention and Control estimates that people with active and untreated tuberculosis may infect 10–15 people per year on average.^40^ By reducing case detection rates, short-term budgetary reductions can increase long-term treatment costs. In the United States of America, tuberculosis budgets were reduced in the aftermath of the 1970s fiscal crisis. There was an initial saving of US$ 100 million but a subsequent outbreak of drug-resistant tuberculosis ultimately cost more than US$ 1 billion to contain.^41^

In conclusion, our analyses provide evidence that recession can lead to short-term reductions in the financial support of programmes for tuberculosis control. The associated decrease in the detection of tuberculosis is projected to result in sustained, long-term rises in tuberculosis incidence, prevalence and mortality.
